# The 5R's of large language model‐assisted diagnosis: A practical framework for hospitalists

**DOI:** 10.1002/jhm.70390

**Published:** 2026-07-03

**Authors:** Peter Barish, Andrew D. Auerbach, Sumant R. Ranji

**Affiliations:** ^1^ Division of Hospital Medicine University of California San Francisco San Francisco California USA; ^2^ Division of Hospital Medicine, San Francisco General Hospital University of California San Francisco San Francisco California USA; ^3^ Division of Clinical Informatics and Digital Transformation University of California San Francisco San Francisco California USA

## Abstract

Diagnostic error remains a major patient safety challenge in hospital medicine. Large language models (LLMs) are increasingly used by clinicians to aid in diagnosis, yet most lack a structured approach for doing so safely and effectively. In this piece, we propose a practical, clinician‐centered framework for LLM‐assisted diagnosis. The 5R's Framework promotes a human‐in‐the‐loop approach that harnesses LLMs to reduce diagnostic error while preserving critical reasoning skills.

## Visual Abstract

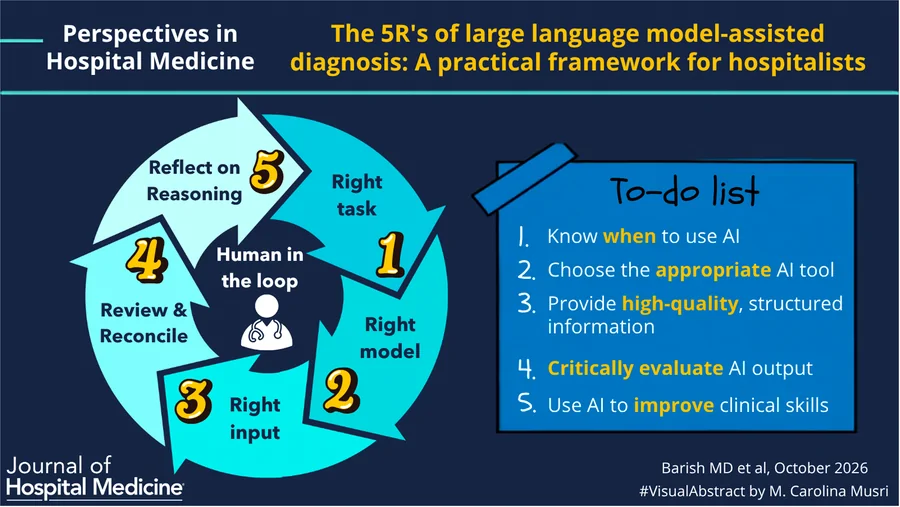

*A hospitalist admits a 70‐year‐old woman with metastatic gastric cancer (on active treatment with paclitaxel) and heart failure with preserved ejection fraction for acute hypoxemic respiratory failure, initially attributed bacterial pneumonia and heart failure exacerbation. Despite antibiotics and diuresis, on hospital day 3 she has escalating oxygen requirements. The hospitalist is uncertain about the diagnosis and wonders if artificial intelligence could assist, but she has not used AI before and is unsure how to proceed*.

## INTRODUCTION

Diagnostic errors occur frequently in hospital medicine,^1^ often resulting from delays in considering or prioritizing a diagnosis, failure to consider complications, or problems with test ordering and interpretation.^2^

Large language models (LLMs) have tremendous promise for improving the diagnostic process. In simulated studies, LLMs have solved complex clinical cases at rates equal to or surpassing expert clinicians.^3^, ^4^, ^5^ Many clinicians are using LLMs to aid in diagnosis; in a 2024 survey, 12% of physicians reported using AI as a diagnostic tool,^6^ a proportion likely increasing due to the availability of free, point‐of‐care applications.

However, there are few studies of LLMs in real‐world clinical care, and LLM use itself has risks.^7^, ^8^ Hospitalists who use LLMs for diagnosis therefore need to be familiar with the strengths and weaknesses of these tools and how to use them as a decision support tool at the bedside.

The “5 rights” framework for clinical decision support systems (CDS) outline principles^9^ to ensure that clinicians receive actionable, evidence‐based information to provide better care. An effective CDSS should deliver the **R**ight information to the **R**ight person in the **R**ight format, using the **R**ight channel and at the **R**ight time in the clinician's workflow. In the same vein, we propose the **5R's of LLM‐assisted diagnosis**, a pragmatic framework grounded in clinical reasoning science. This approach should help hospitalists use LLMs to improve diagnosis while limiting the potential for errors or unintended consequences.

## IMPORTANCE OF A HUMAN‐IN‐THE‐LOOP APPROACH

LLMs are capable, confident, and fast. However, these features can obscure important limitations when applied to clinical care: they can generate plausible sounding but incorrect information (“hallucinations”), inherit biases from their training data, and may lack the nuanced contextual factors that clinicians weigh subconsciously.^7^, ^8^

A primary concern accompanying increasing use of AI, particularly for trainees, is the risk of cognitive deskilling.^10^ If an LLM can instantly generate a thorough differential diagnosis, will the next generation of physicians lose the skill of clinical reasoning?

As AI tools become integrated into diagnosis, we must rigorously reaffirm the clinician's role. LLMs should function as diagnostic assistants, not autonomous diagnosticians. When using an LLM, the cognitive work does not vanish; it shifts. The physician must still perform the critical tasks of data gathering and synthesis to summarize the clinical presentation for the AI and acting on LLM outputs requires a sophisticated understanding of limits and bias. As with all clinical decision support tools, the **physician is ultimately responsible** for their medical decision making.

Thus, an effective “human‐in‐the‐loop” approach is the best way to ensure patient safety, limit liability, and enhance (rather than degrade) clinical reasoning skills.

## THE 5R'S FRAMEWORK

To optimize LLM‐assisted diagnosis, we propose the **5R's framework**. This model guides hospitalists through the safe and effective application of LLMs at the bedside (Figure 1).

1. Right task—Knowing when to use the model—and when to abstain.
  LLMs excel in the creation and prioritization of differential diagnoses, particularly for complex, undifferentiated cases. They can be particularly helpful for expanding a differential when clinicians feel “stuck” or during a diagnostic time‐out. Physicians must start with their own diagnostic thinking (to avoid deskilling), but LLMs can aid in identifying rare diagnoses that might otherwise be overlooked or organize thinking around complex, multi‐system presentations.
  LLMs should be avoided in high‐stakes situations where the risk of model hallucination is unacceptable. This includes time‐sensitive emergencies (e.g., a code blue or rapid response escalation), questions regarding medication dosing, drug‐drug interactions, or specific internal hospital guidelines. In these moments, adherence to peer‐reviewed resources is necessary.
2. Right model—Picking the tool that matches the cognitive job.

**Figure 1:**
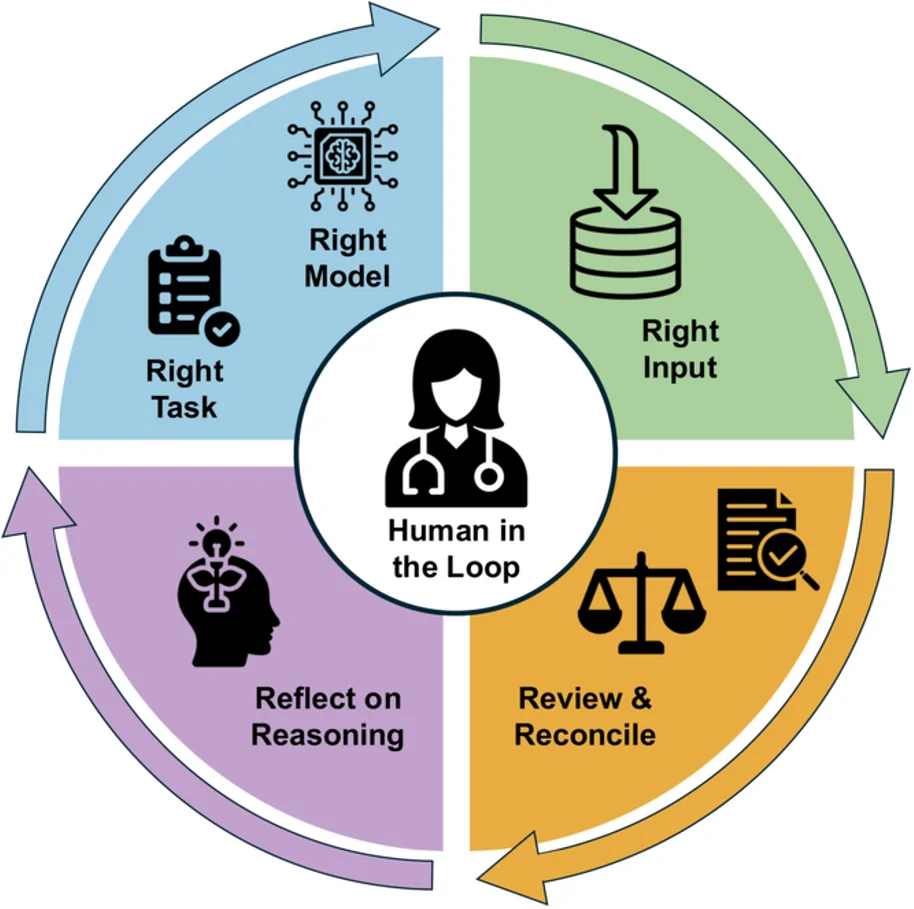
5R's framework for successful large language model‐assisted diagnosis.

Not all LLMs are created equal, and using the wrong architecture for a diagnostic task can lead to suboptimal results. The landscape of high‐performing LLMs (often called “frontier” models) changes rapidly, and hospitalists should be able to distinguish between three broad categories:

1. *Standard models for simple tasks:* For tasks like creating patient education materials, communication scripts, or drafting administrative documents, standard models (e.g., ChatGPT “Instant” or Gemini 2.5 “Fast”) are sufficient, and often free to use.
2. *Reasoning models for logic tasks:* For complex differential diagnosis and multi‐step clinical logic, newer “reasoning” models (e.g., ChatGPT or Gemini with “Thinking” selected) are superior. Reasoning models use “chain‐of‐thought” processing to solve complex problems, mirroring human clinical reasoning.^10^ Emerging evidence also suggests these models are better at medical benchmarks and may be less susceptible to cognitive biases than standard models.^11^, ^12^
3. *Retrieval‐augmented generation (RAG)/“deep research” tools for understanding the evidence:* For specific, detailed knowledge questions (i.e., literature/guideline searches), we recommend tools specifically designed to search and cite medical literature. The most widely used of these is OpenEvidence, which queries an internal database to provide answers backed by specific literature citations. RAG models are very useful for answering knowledge questions but may be less useful for differential diagnosis generation.

3. Right input—Synthesizing data, prompting, and privacy protection.

The quality of the LLM's output is influenced by the quality of the input—the “garbage in, garbage out” principle. This step is also where clinical reasoning is actively practiced.

1. *Structured prompting:* To improve response reliability and quality, we recommend using a structured prompt that defines the **Context** (“assisting with a complex clinical case”), the **Task** (“generate a ranked differential”), and the **Format** (“bullet points, arguments for and against”).

a. *Example:* “I need assistance with a complex Hospital Medicine case. I will give you the case information. Task: please provide a ranked differential diagnosis with key supporting and refuting features for each. Include can't‐miss diagnoses we should consider.”

2. *Data gathering and problem representation:* Before turning to an LLM, the clinician should craft a concise summary of the most salient features of the case (i.e., a Problem Representation), highlighting features that support or reject the working diagnosis. This deliberate reflection, occurring before interacting with the LLM, promotes learning and diagnostic accuracy.^13^, ^14^

a. *Example:* “Mrs. J is an elderly woman with a history of heart failure and gastric carcinoma metastatic to liver and lungs, on paclitaxel, who presented with subacute, progressive, shortness of breath and new hypoxemia. Her admission CXR showed bilateral lower lobe hazy opacities. She has been treated with antibiotics and diuretics for 3 days but has progressively worsening hypoxemia.”

3. *De‐identification:* Strict adherence to institutional policies for protected health information (PHI) is paramount. Clinicians should remove all PHI identifiers (i.e., dates) and generalize ages, unless their institution offers access to a secure LLM that is approved for use with PHI. Information should never be copied from the EHR into any public LLM.

4. Review and reconcile—*Keeping the human in the loop*.
  Once the LLM generates a response, the clinician's role shifts to critical reviewer. Hospitalists should verify specific claims against a trusted reference to check for hallucinations, especially regarding diagnostic criteria or test characteristics.
  Clinicians must also look to mitigate the **risk of bias**. Automation bias—the tendency to favor machine‐generated advice over human judgment—and human cognitive biases within the LLM itself (e.g., anchoring bias, framing effects, hindsight bias), may be at play.^8^ If the LLM agrees with you entirely, ask it to “play devil's advocate” and argue against your top diagnosis. Reconcile the LLMs differential diagnosis with your own: hospitalists should not uncritically accept an LLM's diagnosis but should instead use it to refine diagnostic possibilities and inform further workup.
5. Reflect on Reasoning—Reinforce and calibrate diagnostic skills.

The final “R” ensures that the use of LLMs contributes to the clinician's long‐term growth.^15^ Close each interaction with a distinct **60‐s pause for deliberate reflection**, comparing the LLM's output to your working differential diagnosis.

*Identify discrepancies and calibrate:* If the LLM suggested diagnosis you missed, reflect on *why* you didn't include it. Was it a knowledge gap? Premature closure? This step turns the consult into a learning event (deliberate practice).

*Team‐based learning:* LLM‐assisted diagnosis can also be used for teaching; teams can compare and contrast their differential against the AI's, making the reasoning process visible and collaborative.

## CONCLUSIONS

In the scenario, the hospitalist discussed the case with a colleague; together, they queried an LLM using a deidentified problem representation and structured prompt. The LLM suggested a diagnosis which had not been considered previously: paclitaxel‐induced pulmonary toxicity. After consulting the pulmonary service, the hospitalist initiated treatment with oral prednisone. The patient's symptoms and oxygen requirement improved quickly, and she was discharged on hospital day 7.

This case illustrates the potential of LLMs to improve diagnosis in complex hospitalized patients by offering a “second opinion” on demand. These powerful tools have immense potential for improving diagnosis, but the introduction of AI into the diagnostic process is not without risk. The 5R's Framework aims to promote effective and safe LLM use anchored in the principles of clinical reasoning and deliberate practice. Appropriate LLM use can harness the power of these models to reduce diagnostic error while simultaneously sharpening hospitalists’ own clinical acumen.

## CONFLICT OF INTEREST STATEMENT

The authors declare no conflicts of interest.

## Data Availability

Data sharing is not applicable to this article as no datasets were generated or analyzed during the current study.
